# Non-IG::MYC in diffuse large B-cell lymphoma confers variable genomic configurations and MYC transactivation potential

**DOI:** 10.1038/s41375-023-02134-1

**Published:** 2024-01-06

**Authors:** Chunye Zhang, Ellen Stelloo, Sharon Barrans, Francesco Cucco, Dan Jiang, Maria-Myrsini Tzioni, Zi Chen, Yan Li, Joost F. Swennenhuis, Jasmine Makker, Lívia Rásó-Barnett, Hongxiang Liu, Hesham El-Daly, Elizabeth Soilleux, Nimish Shah, Sateesh Kumar Nagumantry, Maw Kyaw, Mahesh Panatt Prahladan, Reuben Tooze, David R. Westhead, Harma Feitsma, Andrew J. Davies, Catherine Burton, Peter W. M. Johnson, Ming-Qing Du

**Affiliations:** 1 Division of Cellular and Molecular Pathology, Department of Pathology, University of Cambridge, Cambridge, UK; 2 Department of Oral Pathology, Shanghai Ninth People’s Hospital, Shanghai Jiao Tong University School of Medicine, Shanghai, PR China; 3 Cergentis BV, Utrecht, Netherlands; 4 Haematological Malignancy Diagnostic Service, St James’ University Hospital, Leeds, UK; 5 Institute of Clinical Physiology, CNR, Pisa, Italy; 6 East Genomic Laboratory Hub, Cambridge University Hospitals NHS Foundation Trust, Cambridge, UK; 7 Department of Haematology, Hebei General Hospital, Shijiazhuang, PR China; 8 The Haematopathology and Oncology Diagnostic Service, Cambridge University Hospitals NHS Foundation Trust, Cambridge, UK; 9 Cellular Pathology Department, University Hospitals Coventry and Warwickshire NHS Trust, Coventry, UK; 10 Department of Haematology, Norfolk and Norwich University Foundation Hospital, Norwich, UK; 11 Department of Haematology, Peterborough City Hospital, Peterborough, UK; 12 Department of Haematology, James Paget University Hospitals NHS Foundation Trust, Great Yarmouth, UK; 13 East Suffolk and North Essex Foundation Trust, Suffolk, UK; 14 Division of Haematology and Immunology, Leeds Institute of Medical Research, University of Leeds, Leeds, UK; 15 School of Molecular and Cellular Biology, Faculty of Biological Sciences, University of Leeds, Leeds, UK; 16 Southampton NIHR/Cancer Research UK Experimental Cancer Medicine Centre and Southampton Clinical Trials Unit, University of Southampton, Southampton, UK

**Keywords:** B-cell lymphoma, Cancer genetics

## Abstract

*MYC* translocation occurs in 8–14% of diffuse large B-cell lymphoma (DLBCL), and may concur with *BCL2* and/or *BCL6* translocation, known as double-hit (DH) or triple-hit (TH). DLBCL-*MYC*/*BCL2*-DH/TH are largely germinal centre B-cell like subtype, but show variable clinical outcome, with *IG*::*MYC* fusion significantly associated with inferior survival. While DLBCL-*MYC*/*BCL6*-DH are variable in their cell-of-origin subtypes and clinical outcome. Intriguingly, only 40-50% of DLBCL with *MYC* translocation show high MYC protein expression (>70%). We studied 186 DLBCLs with *MYC* translocation including 32 *MYC/BCL2/BCL6*-TH*, 75 MYC/BCL2*-DH and 26 *MYC/BCL6*-DH. FISH revealed a *MYC*/*BCL6* fusion in 59% of DLBCL-*MYC/BCL2/BCL6*-TH and 27% of DLBCL-*MYC*/*BCL6*-DH. Targeted NGS showed a similar mutation profile and LymphGen genetic subtype between DLBCL-*MYC/BCL2/BCL6*-TH and DLBCL-*MYC/BCL2*-DH, but variable LymphGen subtypes among DLBCL-*MYC*/*BCL6*-DH. MYC protein expression is uniformly high in DLBCL with *IG::MYC*, but variable in those with non-*IG::MYC* including *MYC*/*BCL6*-fusion. Translocation breakpoint analyses of 8 cases by TLC-based NGS showed no obvious genomic configuration that enables *MYC* transactivation in 3 of the 4 cases with non-*IG:*:*MYC*, while a typical promoter substitution or *IGH* super enhancer juxtaposition in the remaining cases. The findings potentially explain variable MYC expression in DLBCL with *MYC* translocation, and also bear practical implications in its routine assessment.

## Introduction

Diffuse large B-cell lymphoma (DLBCL) is a group of heterogeneous aggressive B-cell lymphoma with variable cell-of-origin (COO), genetic changes, molecular mechanisms and clinical outcomes. Based on COO, DLBCL can be broadly classified into activated B-cell like (ABC) and germinal centre B-cell like (GCB) subtype, with a subset of the latter further identified as molecular high grade (MHG)/double-hit signature (DHITsig) due to their enriched *MYC* expression and centroblast signatures [1, 2]. Based on genetic alterations, DLBCL can be subdivided into distinct subgroups using LymphGen algorithm or other: MCD (*MYD88*^L265*P*^ and *CD79B* mutations), N1 (*NOTCH1* mutation), A53 (aneuploidy with *TP53* inactivation), BN2 (*BCL6* translocation and *NOTCH2* mutation), ST2 (*SGK1* and *TET2* mutated) and EZB (*EZH2* mutation and *BCL2* translocation), with the latter subgroup further divided into EZB-MYC+ and EZB-MYC- according to MYC signature [3, 4]. There is a broad correlation between COO molecular subtypes and genetic subgroups. ABC-DLBCL largely comprises of MCD, N1 and A53, while GCB-DLBCL is primarily composed of EZB and ST2, with BN2 seen in both ABC and GCB-DLBCL. These subgroups are further underpinned by their distinct molecular mechanisms and different clinical outcomes.

Despite the steady progress in molecular characterization and sub-classification of DLBCL, few of these advances are applied in a routine clinical setting. For routine diagnosis and prognostication of DLBCL, only *MYC*, *BCL2* and *BCL6* translocations are investigated along with international prognostic index. *MYC* translocation occurs in 8–14% of DLBCL. This translocation can occur together with *BCL2* and/or *BCL6* translocation, known as double-hit (DH) or triple-hit (TH). Among *MYC* translocation positive DLBCL, ~9% are *MYC/BCL2/BCL6*-TH, ~40% and ~18% are *MYC*/*BCL2*-DH and *MYC*/*BCL6*-DH respectively [5–7]. Most of cases with *MYC*/*BCL2-*DH/TH are GCB subtype or EZB-MYC+ [3, 5]. In contrast, those with *MYC*/*BCL6*-DH are rather heterogeneous in their molecular subtypes, with 30% each being GCB or ABC subtype respectively, 15% due to MHG, and the remaining cases unclassifiable [5]. These cases showed a mutation profile remarkably different from those with *MYC*/*BCL2-*DH/TH, but do not exhibit any prominent signatures although a proportion of these cases are associated with *NOTCH2* mutation, thus BN2 subtype [5]. For these reasons, the 5th edition of the World Health Organization Classification of Haematolymphoid Tumours (WHO-HAEM5) excludes the cases with concomitant *MYC* and *BCL6* rearrangements (without *BCL2* rearrangement) from the DH entity and renames the entity as diffuse large B-cell lymphoma/high-grade B-cell lymphoma with *MYC* and *BCL2* rearrangements (DLBCL/HGBL-*MYC*/*BCL2*) to recognise their variable morphology [8].

The clinical outcome of DLBCL/HGBL-*MYC*/*BCL2*-DH is also heterogeneous. Cases with *IG::MYC* are significantly associated with worse progression-free survival (PFS) and overall survival (OS), particularly within the first two years of diagnosis, while those with non-*IG::MYC* showed no significant difference in both PFS and OS from DLBCL without *MYC* translocation [7, 9]. The molecular mechanisms underlying the different clinical impacts by *MYC* translocation partner are unclear. In addition, MYC protein expression varies considerably in DLBCL with *MYC* translocation, ranging from negative to 100% positivity in lymphoma cells [10–12]. In DLBCL with *IGH*::*MYC*, the breakpoint commonly occurs in region spanning the 5’UTR and intron 1 of the *MYC* gene and the switch region of the *IGH* locus respectively, thus placing the *MYC* gene in close proximity of the highly active *IGH* super enhancer, causing *MYC* constitutive over-expression [13]. Moreover, DLBCL with *IGH*::*MYC* often acquire *MYC* mutations that impair MYC protein degradation, consequently sustaining its expression and function [5]. However, the impact of non-*IG* partner on *MYC* expression is unclear. Among the known non-*IG* partners of *MYC* translocation including *BCL6*, *ZCCHC7* and *RFTN1*, *BCL6* is the most frequent [13, 14]. It also remains unclear how often non-*IG*::*MYC* translocation involves *BCL6* as a partner, and how non-*IG*::*MYC* impacts on *MYC* activation given their clear difference in clinical impact from the *IG*::*MYC* translocation. To investigate these, we studied 186 cases of DLBCL with *MYC* translocation including 32 *MYC/BCL2/BCL6*-TH*, 75 MYC/BCL2*-DH and 26 *MYC/BCL6*-DH by combined analyses of *MYC* translocation partner and MYC protein expression, mutation profiling and breakpoint analysis of *MYC* translocation in selected cases to understand their transactivation potential.

## Materials and methods

The study was performed in accordance with local ethical guidelines for the research use of tissue materials with the approval of the ethics committees of the involved institutions (05-Q1604-10, 04-Q1205-125, 10-H0504-79).

**Fig. 1:**
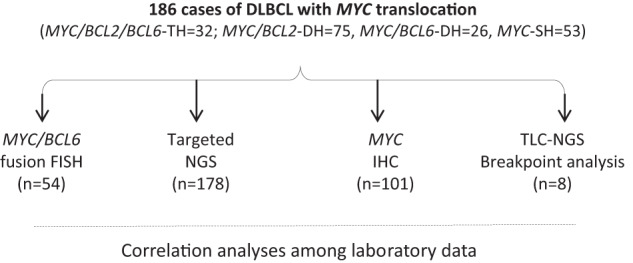
Summary of DLBCL with *MYC* translocation and experiments carried out.

A total of 186 cases of DLBCL with *MYC* translocation were retrieved from surgical files of Addenbrookes Hospital, University of Cambridge and HMDS, St James’ University Hospital, Leeds, UK. These cases comprised of 32 *MYC/BCL2/BCL6*-TH, 75 *MYC/BCL2*-DH, 26 cases with *MYC*/*BCL6*-DH, and 53 cases *MYC*-single hit (SH) (Fig. 1).

### Interphase fluorescence in situ hybridisation (FISH)

Chromosome translocation status at the *MYC*, *BCL2* and *BCL6* locus was available from routine haematopathological diagnosis or previous studies [5]. Further interphase FISH with *MYC*/*BCL6* (Cytocell), *MYC*/*IGH* (Abbott), *MYC*/*IGK* and *MYC*/*IGL* (Cytocell) dual fusion probes were performed on FFPE tissue slides where indicated in the present study.

### Immunohistochemistry

MYC (Abcam clone Y69) and BCL6 (Leica Clone LN22) immunohistochemistry were performed where possible in all cases where tissue materials remained available using the Bond-III system (Leica Biosystems) with the Bond Polymer Refine Detection Kit as the same condition of routine histopathological diagnosis. This was carried out centrally in the Cambridge lab and the staining intensity (weak, moderate, strong) and percentage in tumour cells (>70% or <70%) were scored [11].

### DNA extraction and quality assessment

Histology was reviewed and areas containing confluent lymphoma cells (>40%) in each specimen were microdissected on consecutive tissue sections. DNA was extracted using the QIAamp DNA Micro Kit (QIAGEN, Crawly, UK), quantified with a Qubit® Fluorometer (Life Technologies, UK) and assessed for quality by PCR [5, 15].

### Mutation analysis by targeted sequencing

The mutation data in 125 cases were from a previous study, in which a panel of B-cell lymphoma associated genes (*n* = 70) were sequenced using HaloPlexHS target enrichment and Illumina HiSeq4000 platform, with a well-validated in house variant calling pipeline [5]. In 53 cases, mutation data were similarly obtained but using TWIST capture target enrichment of a much larger gene panel (*n* = 191) (Table S1) [16].

LymphGen genetic subtypes were assigned where possible according to Wright et al [3].

### Targeted locus capture next generation sequencing (TLC-NGS)

TLC-NGS was essentially carried out as previously described [17]. FFPE tissue sections were deparaffinised, followed by a 30 min pretreatment step at 90 °C, digestion with NlaIII restriction enzyme and ligation with T4 DNA ligase. The sample was incubated at 80°C overnight to reverse crosslinking and then subjected to DNA purification. A total of 100 ng DNA was fragmented and used for NGS library preparation, hybridization with capture probes using Roche HyperCap reagents according to the manufacturer’s instructions. Paired-end sequencing was performed using an Illumina Novaseq 6000. TLC-NGS reads were mapped to the human genome (hg19) using BWA-MEM (version: 0.7.17-r1188; settings: -SP -k12 -A2 -B3) in paired-end mode, and gene rearrangements were identified using PLIER (Proximity-Ligation based IdEntification of Rearrangements) according to previously validated pipeline [17].

### Statistical analysis

Associations among *MYC* translocation, translocation partner and MYC protein expression were analysed using the Fisher’s exact test. All quoted *P* values are two-sided.

## Results

### *BCL6* frequently involves *MYC* translocation in DLBCL with *MYC*/*BCL6*/*BCL2*-TH or *MYC*/*BCL6*-DH

**Fig. 2:**
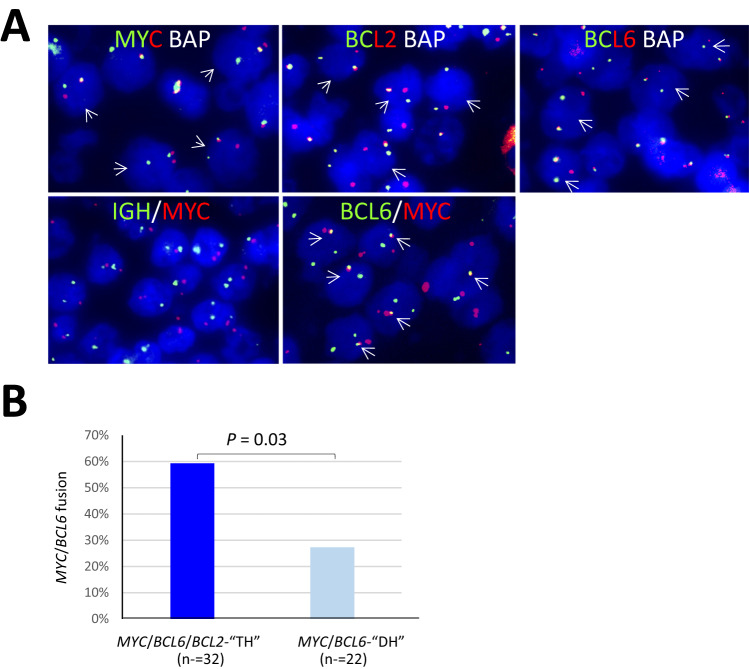
*MYC*/*BCL6* fusion accounts for a high proportion of DLBCL with *MYC* and *BCL6* translocation. **A** Example of interphase FISH in a case with a triple hit (TH), in which the *MYC* and *BCL6* translocation detected by their breakapart probes (BAP) are due to *MYC*/*BCL6* fusion. **B** The frequency of *MYC*/*BCL6* fusion is significantly higher in cases with *MYC*/*BCL6*/*BCL2*-”TH” than those with *MYC*/*BCL6* “double hit” (DH).

Interphase FISH with the *BCL6*/*MYC* fusion probe was performed in 54 cases of DLBCL with *MYC*/*BCL2*/*BCL6-*TH (*n* = 32) or *MYC*/*BCL6-*DH (*n* = 22). Among these cases, 25 (46.3%) had evidence of genomic fusion between the *MYC* and *BCL6* loci by FISH (Fig. 2A), and the frequency of *MYC*/*BCL6* fusion was significantly higher in the *MYC/BCL2/BCL6*-TH (19/32 = 59%) than the *MYC/BCL6*-DH (6/22 = 27%) group (Fig. 2B).

Among the 25 cases with FISH evidence of *MYC*/*BCL6* fusion, 24 had complete data on *IG*/*MYC* fusion by interphase FISH with the *MYC/IGH* fusion probe, and additional *MYC*/*IGK*(*L*) fusion probe if no evidence of *MYC/IGH* fusion. Six of these cases had an *IGH*::*MYC* fusion, and this is consistent with previous observation of a three way translocation involving the *MYC*, *BCL6* and *IGH* loci by cytogenetic studies [18].

### Genetic features of DLBCL with *MYC/BCL6* fusion

Mutation profiling by targeted NGS was carried out in 178 cases, and 135 of these cases were successfully subtyped using the LymphGen algorithm [3].

**Fig. 3:**
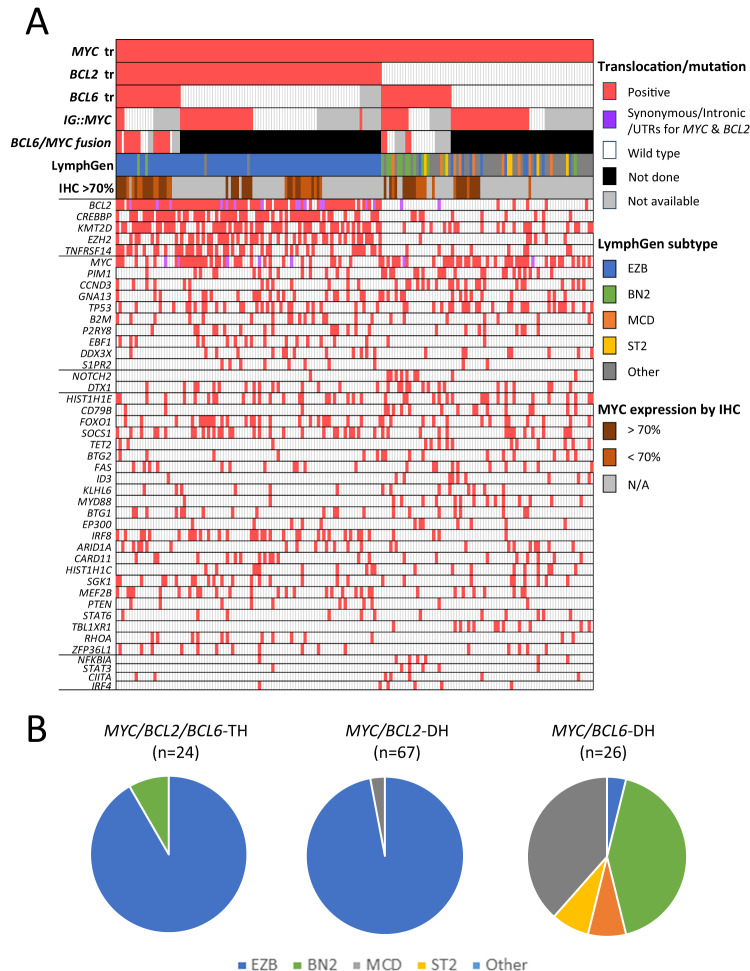
Mutation profile (**A**) and LymphGen genetic subtype (**B**) according to translocation status. tr translocation, IHC immunohistochemistry, TH triple hits, DH double hits.

Overall, the mutation profile of the *MYC*/*BCL2*/*BCL6*-TH group is very similar to that of the *MYC*/*BCL2*-DH group (Fig. 3A), characterised by frequent mutations in follicular lymphoma associated genes (*BCL2*, *CREBBP*, *KMT2D*, *EZH2, TNFRSF14*). Our previous study shows that most cases with *MYC*/*BCL2*/*BCL6*-TH, like those with *MYC*/*BCL2*-DH, are GCB, with a subset being MHG [5]. In support of this, the present study further demonstrated that both *MYC*/*BCL2*/*BCL6*-TH (22/24 = 92%) and *MYC*/*BCL2*-DH (65/67 = 97%) groups were predominantly the EZB-MYC+ genetic subtype. Within the *MYC*/*BCL2*/*BCL6*-TH group, there were no apparent differences in the mutation profile and LymphGen genetic subtype between *MYC*/*BCL6* fusion positive and negative cases (Fig. 3A).

In contrast, the mutation profile of DLBCL-*MYC*/*BCL6*-DH was of less characteristic, but clearly differed from that of the *MYC*/*BCL2*/*BCL6*-TH or *MYC*/*BCL2*-DH group (Fig. 3A). The *MYC*/*BCL6*-DH cases vary in their COO subtype as shown in our previous study [5]. The present study further demonstrated that these cases varied in their LymphGen genetic subtypes although more frequently being the BN2 subtype or unclassifiable (Fig. 3B). Within the *MYC*/*BCL6*-DH group, there were also no apparent differences in the mutation profile and genetic subtype between *MYC*/*BCL6* fusion positive and negative cases albeit based on few cases.

### MYC protein expression is uniformly high in cases with *IG*::*MYC* but varies in those with non-*IG*::*MYC*

Given that *MYC* translocation is thought to dysregulate its transcription control, we compared MYC protein expression according to *MYC* translocation partner. High MYC expression was defined when the protein is expressed in 70% of lymphoma cells with moderate to strong staining by immunohistochemistry as such high MYC protein expression has been previously shown to identify high risk cases [11].

**Fig. 4:**
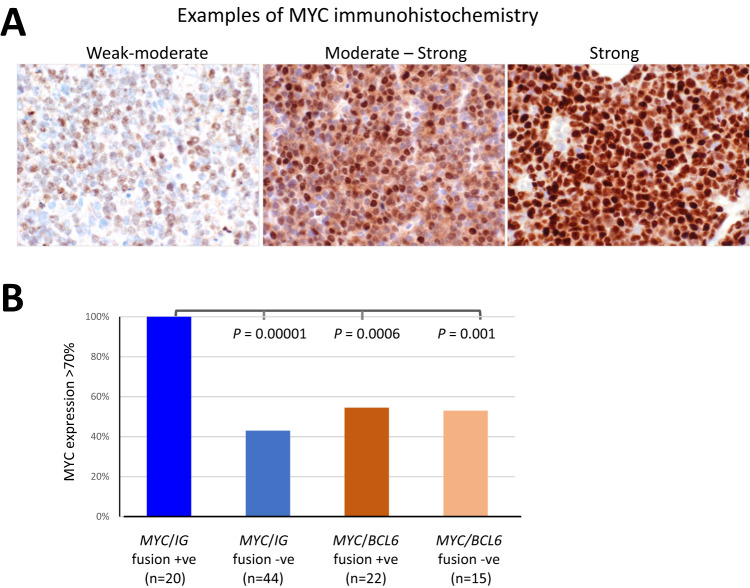
MYC protein expression and its correlation with *MYC* translocation partner. **A** Examples of MYC immunohistochemistry and grading; (**B**) high MYC protein expression is invariably seen in DLBCL with *IG*::*MYC*, but only in up to 50% cases with non-*IG*::*MYC* translocation..

High MYC protein expression was seen in each of the 20 cases of DLBCL with *IG*::*MYC* translocation investigated (Fig. 4). Among DLBCL with non-*IG*::*MYC* translocation including those with *MYC*/*BCL6* fusion, MYC expression was variable, with only up to 50% cases showing a high MYC protein expression (Fig. 4B). There was no difference in the proportion of cases with high MYC protein expression between the *MYC*/*BCL6* fusion positive and negative groups (Fig. 4). These findings suggest that non-*IG*::*MYC* translocations may have variable effects on *MYC* transcription control and not every non-*IG*::*MYC* translocation can cause constitutive *MYC* expression.

### Breakpoint analysis of *MYC* translocation reveal insights explaining variable MYC expression

To investigate why MYC protein expression was variable in cases with non-*IG*::*MYC* translocation, we performed TLC-NGS and breakpoint analyses in 8 cases, including 4 with non-*IG::MYC* (3 with *MYC*/*BCL6* fusion) and 4 with *IGH::MYC* respectively. In each case, TLC-NGS investigation confirmed the findings of FISH analyses, and importantly unravelled the breakpoints and orientation of the involved genes, thus helping to understand their transcriptional potential (Table 1).

**Table 1:** Detection of chromosome translocation by TLC-NGS.

Targets*	*BCL2*	*BCL6*	M*Y*C	*IGH*
Case				
DLBCL-134	*IGH*	MYC	*BCL6*	*BCL2*
DLBCL-173	*IGH*	*MYC*	*BCL6*	*BCL2*
DLBCL-136	*IGH*	chr16 (*CIITA*, intron 1)	chr8 (*TOX*, intron 1)	*BCL2*
DLBCL-123		*IGH*, *MYC*	*BCL6, IGH*	*BCL6, MYC*
DLBCL-154	*IGH*, BCL6	*BCL2*, *IGH*; chr3 (no genes annotated)	chr12 (*HNRNPA1*, intron 1)	*BCL2, BCL6*
LO318	*IGH*, chr17 (~55 Mb)	chr4 (~40 Mb)	*IGH*	*BCL2, MYC*
DLBCL-96		*IGH*	*IGH*	*BCL6, MYC*
DLBCL-178		chr13 (*LCP1*, intron 1)	chr14 (~69 Mb)	

^*^Various targets captured by the TLC-NGS design, while the fusion partners identified are shown in the corresponding cell in each case.

**Fig. 5:**
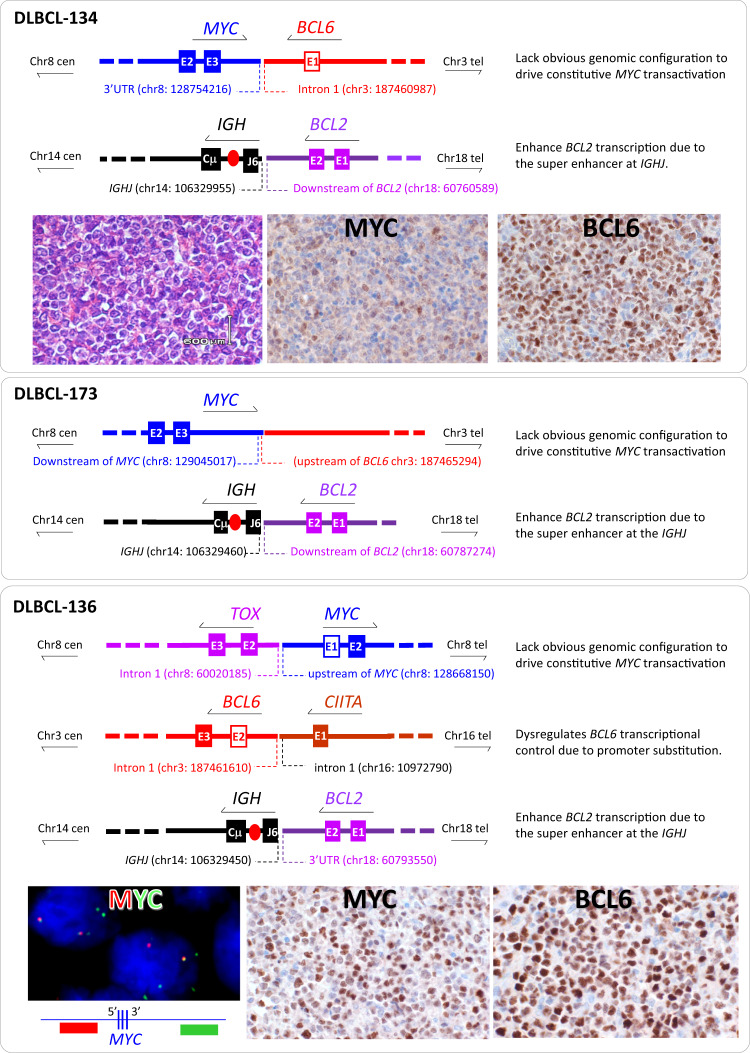
*MYC* translocation with *BCL6* or other partners lacks genomic configuration that activates *MYC* transcription. Genomic breakpoint sequencing analyses was performed by targeted locus capture-based next generation sequencing (TLC-NGS) with sequence annotations based on human genome (hg19). E: exon (fill box: coding exon; non-filled box: non-coding exon). Cen: centromere; Tel: telomere.

**Fig. 6:**
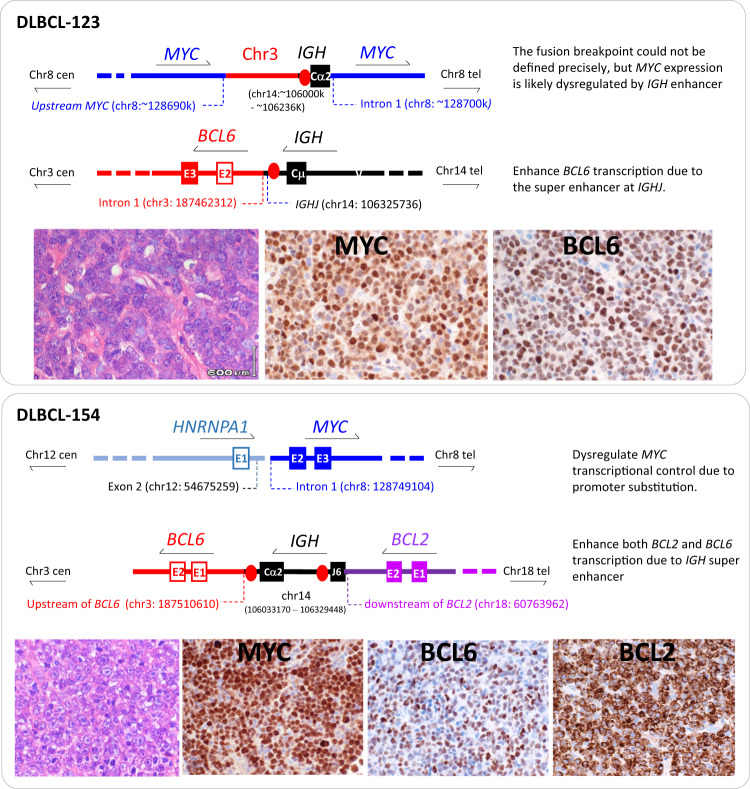
*MYC* translocation with *IGH* or novel partner confers genomic configuration that activates* MYC* transcription. Genomic breakpoint sequencing analyses was performed by targeted locus capture-based next generation sequencing (TLC-NGS) with sequence annotations based on human genome (hg19). E: exon (fill box: coding exon; non-filled box: non-coding exon). Cen: centromere; Tel: telomere.

Among the three cases with *MYC*/*BCL6* fusion, two (DLBCL-134, DLBCL-173) involved direct juxtaposition between the *MYC* and *BCL6* loci, with the breakpoints occurring downstream or at the 3ʹUTR of the *MYC* gene, but upstream or within the intron 1 of the *BCL6* gene (Fig. 5). In both cases, the rearranged *MYC* and *BCL6* genes were in an opposite orientation, thus no structural changes in the 5ʹ region of *MYC* transcriptional control albeit uncertain on any potential effect of the super enhancers downstream of the *MYC* and also at the 5ʹ region of the *BCL6* gene [19, 20]. In both cases, the *MYC* protein expression was weak in <40% lymphoma cells. In the remaining case with *MYC*/*BCL6* fusion (DLBCL-123), an insertion of a segment of chromosome 3 sequence neighbouring to the *BCL6* locus together with a segment of the *IGH* switch region occurred within the intron 1 of the *MYC* gene (Fig. 6). Although the precise breakpoints of the inserted *IGH* sequence could not be accurately defined, the involved region spanned the switch super enhancer, which could potentially drive *MYC* expression. In keeping with this, *MYC* protein was strongly expressed in most lymphoma cells in this case (Fig. 6).

Among the 5 cases without *MYC/BCL6* fusion by FISH, TLC-NGS analyses confirmed the FISH observations in each case, and further identified their translocation partners (Table 1). Two cases showed a novel *MYC* translocation: one fused with *TOX* at 8q12 in an opposite orientation (DLBCL-136), the other fused with *HNRNPA1* at 12q13 in the same orientation (DLBCL-154) (Figs. 5, 6). In both cases, the *MYC* breakpoint occurred either upstream (in the case with *TOX*) or in the intron 1 (in the case with *HNRNPA1*) of the *MYC* gene. In the case of *TOX*/*MYC* fusion, *MYC* transcription was unlikely driven directly by the *TOX* gene as the translocated *TOX* was in opposite orientation with *MYC* and loose its 5’ transcriptional regulatory region, but MYC protein expression was moderately high. Interestingly, both TLC-NGS and interphase FISH in this case showed increased copies of both the rearranged (3–6 copies by interphase FISH) and non-rearranged (2 copies by interphase FISH) *MYC* alleles, in keeping with the variable staining extensity among lymphoma cells (Figs. 5, S1). In the case with *HNRNPA1::MYC* fusion, *MYC* was in the same orientation with *HNRNPA1*, and placed under the transcription control of *HNRNPA1*. *HNRNPA1* encodes a heterogeneous nuclear ribonucleoprotein that is ubiquitously expressed, and strong *MYC* protein expression was uniformly seen in lymphoma cells of this case (Fig. 6).

In the remaining three cases (L0318, DLBCL-96, DLBCL-178) without *MYC*/*BCL6* fusion, *MYC* translocation was associated with *IGH* (Table 1).

**Fig. 7:**
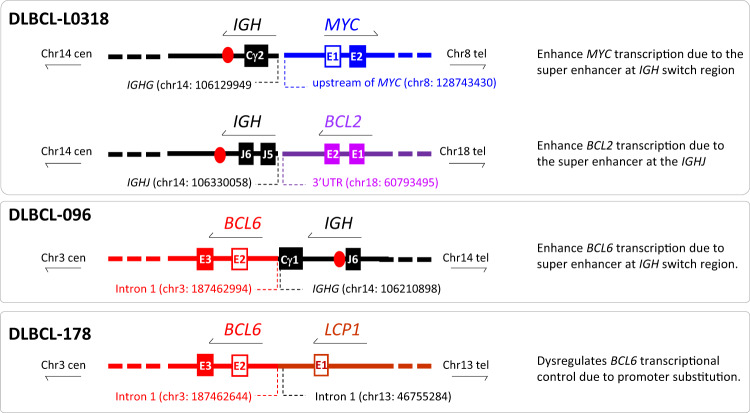
*MYC* translocation with *IGH* or novel partner confers genomic configuration that activates *MYC* transcription. Genomic breakpoint sequencing analyses was performed by targeted locus capture-based next generation sequencing (TLC-NGS) with sequence annotations based on human genome (hg19). E: exon (fill box: coding exon; non-filled box: non-coding exon). Cen: centromere; Tel: telomere.

Apart from the above novel *MYC* translocations, TLC-NGS also identified previously known *LCP1*::*BCL6* (DLBCL-178, Fig. 7) and *CIITA*::*BCL6* fusion each in one case (DLBCL-136, Fig. 5). In both cases, the genomic fusion was in the same orientation and the breakpoint was in the intron 1 of both *BCL6* gene and its partner gene, and these genomic configurations are typical of *BCL6* promoter substitution by its translocation which causes enhanced BCL6 expression (Figs. 5, 7). In the case with *HNRNPA1::MYC* fusion (DLBCL-154), TLC-NGS revealed additionally a complex fusion among *BCL6*, *IGH* and *BCL2* (Fig. 6), with the *IGH* segment (from the joining to the switch region) in between the *BCL6* and *BCL2* gene on derivative chromosome 3. In this case, the presence of *IGH* super enhancers (at both joining and switch region) most likely drive constitutive *BCL6* and *BCL2* transactivation, hence the strong expression of both proteins in lymphoma cells (Fig. 6).

## Discussion

The present study reports several significant novel findings, and they include: (1) *MYC* and *BCL6* translocation in a significant proportion of DLBCL, particularly those with *MYC/BCL2/BCL6*-TH, are due to a direct juxtaposition between the *MYC* and *BCL6* loci, rather than being an independent event; (2) MYC protein expression is uniformly high in DLBCL with *IG*::*MYC*, but varies in those with non-*IG::MYC*, including *BCL6/MYC* fusion; (3) *MYC* translocation with non-*IG* partner may not always acquire a genomic configuration that enables *MYC* constitutive transactivation, resulting in high MYC expression. These findings provide molecular insights, which explain several perplexing features of DLBCL with *MYC* translocation, and also bear practical implications in routine prognostic assessment.

*MYC* and *BCL6* translocation detected by interphase FISH with their respective break-apart probes was commonly referred as independent oncogenic events, thus recorded as DH or TH when additional *BCL2* translocation is present. Remarkably, 59% of the so-called *MYC*/*BCL2*/*BCL6-*TH and 27% of *MYC*/*BCL6-*DH DLBCL are actually due to a direct genomic fusion between the *MYC* and *BCL6* loci. The finding is not totally unexpected as *MYC* is one of the many promiscuous translocation partners of *BCL6*, and t(3;8)(q27;q24)/*BCL6*::*MYC* and t(3;8;14)(q27;q24;q32)/*IGH*::*BCL6*/*MYC* have been previously reported [18, 21].

A major molecular mechanism underpinning the oncogenic potential of *MYC* translocation is its transactivation due to juxtaposition to a super enhancer, such as those at the *IGH* joining and switch region or promoter substitution. The *IGH* super enhancers are expected to be highly active in all mature B-cells as they express high levels of immunoglobulin. Such super-enhancer mediated transcriptional activation, unlike promoter substitution, is independent of the genomic orientation of the *MYC* and *IG* genes and to a certain extent also of the “linear” distance between the two genes [22], thus explaining the uniform high MYC protein expression seen in DLBCL with *IG::MYC*, and also Burkitt lymphoma.

Among the 4 cases of DLBCL with non-*IG::MYC* investigated by TLC-NGS, 3 showed *MYC* gene in an opposite orientation with its translocation partner (*BCL6*, *TOX*), without affecting the *MYC* promoter region. The moderate variable MYC expression in the case with *TOX::MYC* (DLBCL-136) is most likely the result of *MYC* gene amplification (Fig. 5, Fig. S1). Otherwise, there was no evidence of constitutive MYC expression in these cases. There were potential super enhancers downstream of the *MYC* gene and in the translocated *BCL6* region [19, 20], the potential impact on these super enhancers by these translocations is unclear. As the transactivation potential of super enhancers depend on cell type and differentiation stage and is regulated by a range of factors, such as genetic/epigenetic modifications and transcriptional factor binding [20, 23, 24], different translocations may give rise to variable potentials of *MYC* transactivation, from low levels of dysregulation to utmost constitutive activation. Nonetheless, lack of high MYC expression in these cases suggests these translocations do not cause *MYC* constitutive transactivation. This speculation is in keeping with the previous observation that a proportion of DLBCL with *MYC* translocation lack high *MYC* mRNA and protein expression [11, 12]. In contrast, the remaining case (DLBCL-154) with *HNRNPA1::MYC* is a typical promoter substitution, and shows strong uniform MYC expression as expected since *HNRNPA1*, encoding for an RNA binding protein, is ubiquitously expressed (Fig. 6).

The above findings potentially explain why *IGH::MYC*, but not non-*IG::MYC* confers significantly inferior survival in patients with DLBCL-*MYC/BCL2*-DH [7], and also why ~25% of DLBCL with *MYC* translocation, including those with a *MYC/BCL2*-DH, are conventional GCB, but not MHG subtype [1]. Our observations also highlight the heterogeneous MYC expression in DLBCL with non-*IG::MYC* translocation. Of note, 44% of DLBCL with non-*IG::MYC*/*BCL2*-DH lacked high MYC protein expression above 70% (Fig. 3A). It remains to be investigated whether there is any potential difference in clinical outcome between *non-IG::MYC* translocation positive DLBCL with high and low MYC protein expression, and whether those with high MYC expression are similar to cases with *IG::MYC* in their clinical outcome. To address this pivotal question, a large cohort of genetic subtype matched DLBCL with *MYC* translocation, such as those with *MYC/BCL2*-DH, is required.

In DLBCL, *MYC* and *BCL6* translocation are most likely acquired due to relentless exposure to somatic hypermutation and class switch activities during B-cell expansion in germinal centres, and are likely a secondary event [13, 25]. This is particularly evident in cases with *BCL2* translocation, which is the primary genetic event, occurring as a consequence of erroneous VDJ recombination at the pre-B stage of B-cell development in the bone marrow. The secondary structural changes may not be always a driver event, similar to the point mutations in many well-known lymphoma genes acquired due to somatic hypermutation activities [26]. In view of this and the above discussion, it is pertinent to question whether every non-*IG*::*MYC* translocation in DLBCL is an activation event, albeit to be attested in future studies.

In routine clinical practice, interphase FISH is used for detection of *MYC*, *BCL2* and *BCL6* translocation, together with their translocation partners, although commonly only including *IGH*. Among *MYC* translocation positive DLBCL, *IG*::*MYC* accounts for ~55% of cases [7, 9]. The full spectrum of non-*IG* partners of *MYC* translocation remains to be characterised although *BCL6* may account for a majority. A major challenge to delineate whether a non-*IG*/*MYC* translocation is a constitutive activation event, thus clinically important, is to characterise its genomic configuration, search for evidence that enables *MYC* constitutive transactivation. This cannot be resolved by interphase FISH even when the translocation partner is known, but requires breakpoint analyses such as by TLC-NGS which is not yet available in a routine clinical setting. In the absence of any knowledge of genomic configuration of the translocation, the pathogenic potential and the prognostic value of non-*IG*/*MYC* translocation need to be interpreted in conjunction with MYC protein expression.

Our findings also raise the debate whether all DLBCL should be investigated for *MYC* translocation with regard to risk stratification in routine histopathological diagnosis by interphase FISH or first screened by MYC immunohistochemistry (where necessary immunohistochemistry with an alternative antibody to rule out potential false negative due to mutation impairing the antibody binding site [12]), and only cases with MYC protein expression above a certain level (to be determined) selected for further FISH analyses. Further breakpoint analysis of non-*IG*/*MYC* translocation and their correlation with the level of MYC protein expression in a large cohort should help to resolve these practical issues. Nonetheless, it is important to routinely investigate whether *MYC* translocation is associated with *IG* (both heavy and light chain) loci and MYC protein expression as both have been shown to be associated with adverse clinical outcome.

In summary, a significant proportion of DLBCL with both *MYC* and *BCL6* translocations are due to direct juxtaposition between the two genomic loci. *MYC* translocation involving non-*IG* loci including *BCL6* varies in their genomic configurations, and may not often gain genomic configuration that can cause constitute *MYC* transactivation, leading to its enhanced protein expression. The prognostic value of *MYC* translocation needs to be interpreted in conjunction with its translocation partner and MYC protein expression level.

### Supplementary information

Supplementary figure S1

Supplmentary Table S1

## Data Availability

All core data generated or analysed during this study are included in this published article, and additional raw data are available from the corresponding author on reasonable request.
