# Supplementary material for: Non-IG::MYC in diffuse large B-cell lymphoma confers variable genomic configurations and MYC transactivation potential
Source: Leukemia. 2024 Jan 6;38(3):621–9. doi: 10.1038/s41375-023-02134-1 (PMC10912016; doi:10.1038/s41375-023-02134-1)
Supplement: Supplementary file 1 — Supplementary figure S1 [file 41375_2023_2134_MOESM1_ESM.pptx]

## Slide 1
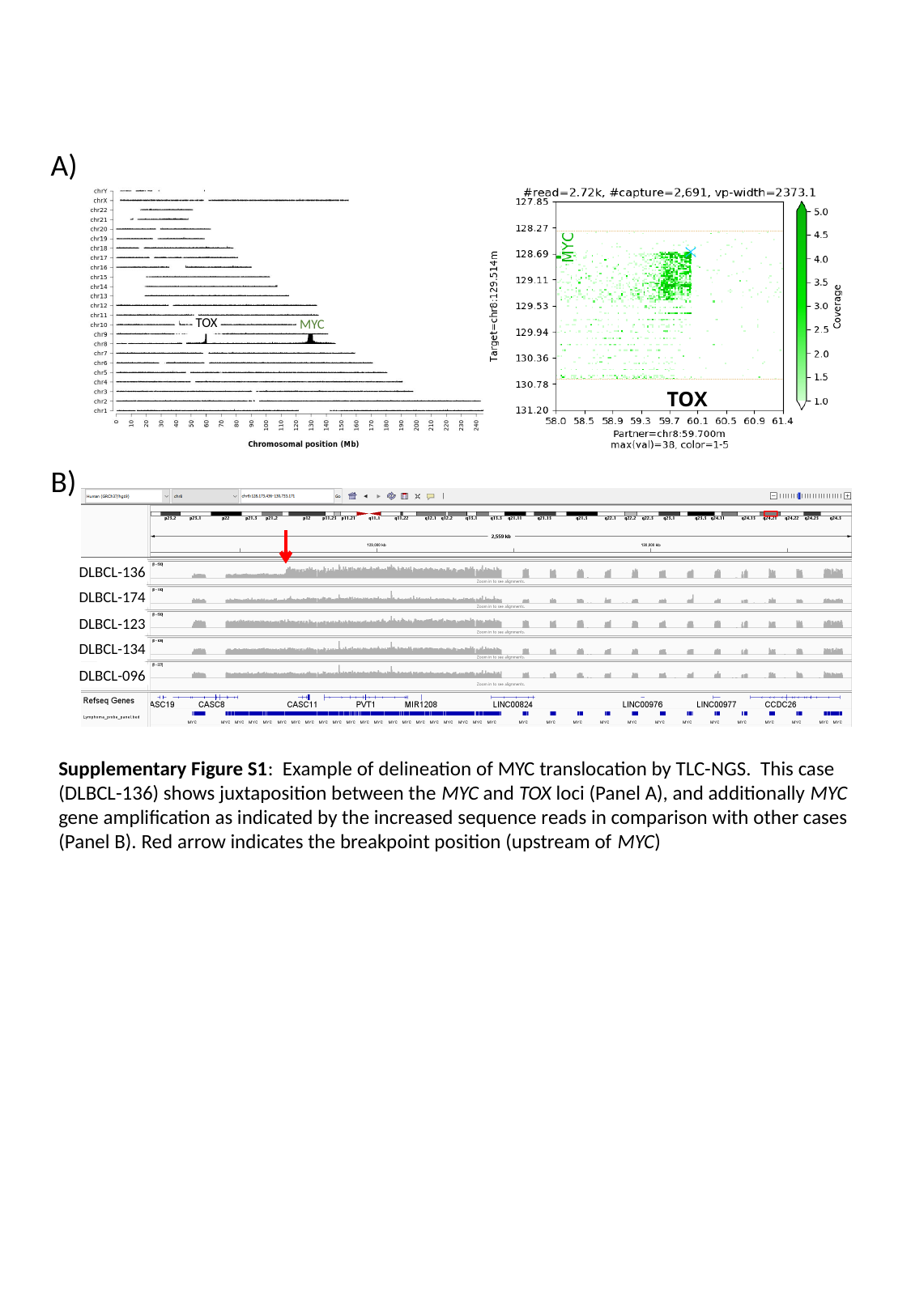

A)
TOX
TOX
MYC
B)
DLBCL-136
DLBCL-174
DLBCL-123
DLBCL-134
DLBCL-096
Supplementary Figure S1: Example of delineation of MYC translocation by TLC-NGS. This case (DLBCL-136) shows juxtaposition between the MYC and TOX loci (Panel A), and additionally MYC gene amplification as indicated by the increased sequence reads in comparison with other cases (Panel B). Red arrow indicates the breakpoint position (upstream of MYC)
