# Supplementary material for: Non-IG::MYC in diffuse large B-cell lymphoma confers variable genomic configurations and MYC transactivation potential
Source: Leukemia. 2024 Jan 6;38(3):621–9. doi: 10.1038/s41375-023-02134-1 (PMC10912016; doi:10.1038/s41375-023-02134-1)
Supplement: Supplementary file 2 — Supplmentary Table S1 [file 41375_2023_2134_MOESM2_ESM.docx]

**Supplementary Table S1: Gene panel investigated by targeted next generation sequencing.**

| ABCA10 | CUX1 | HVCN1 | NRAS | TBL1XR1 |
| --- | --- | --- | --- | --- |
| ABCA6 | CXCR4 | IBTK | OSBPL10 | TCF3 |
| ADGRL2 | CYLD | ID3 | P2RY8 | TCF4 |
| ARID1A | DDX10 | IFI44 | PAPOLG | TET2 |
| ARID1B | DDX3X | IGLL5 | PAX5 | TFEB |
| ARID4B | DDX46 | IL10RA | PDCD1LG2 | TFPT |
| ARID5B | DDX5 | ILF3 | PERP | TLR2 |
| ATM | DNMT3A | IRF4 | PIK3CD | TMEM30A |
| B2M | DTX1 | IRF8 | PIM1 | TMSB4X |
| BCAT1 | DUSP2 | ITPKB | PIM2 | TNFAIP3 |
| BCL10 | EBF1 | KLF2 | PLCG2 | TNFRSF14 |
| BCL11A | EP300 | KLHL14 | POU2AF1 | TNFSF13B |
| BCL2 | ERCC5 | KLHL2 | POU2F2 | TNFSF14 |
| BCL6 | ETS1 | KLHL6 | PPM1D | TOX |
| BCL7A | ETV6 | KMT2D | PRDM1 | TP53 |
| BRAF | EZH2 | KRAS | PRDM15 | TRAF3 |
| BTG1 | FAS | LPP | PRKCB | TRRAP |
| BTG2 | FBXW7 | LTB | PTEN | UBE2A |
| BTK | FNDC3A | MALT1 | PTPN6 | WEE1 |
| C2CD2 | FOXO1 | MAP2K1 | PVT1_promoter | WISP1 |
| C4B_2 | FOXO3 | MCL1 | QTRT1 | XPO1 |
| CA8 | FXYD6 | MEF2B | RB1 | ZC3H12A |
| CARD11 | GNA13 | MIB2 | RCOR1 | ZDHHC18 |
| CCND3 | GRB2 | MIR17HG | REL | ZEB2 |
| CD19 | GTDC1 | MLLT3 | RELB | ZFAND5 |
| CD274 | HIST1H1B | MPEG1 | RHOA | ZFHX3 |
| CD58 | HIST1H1C | MTOR | RRAGC | ZFP36L1 |
| CD70 | HIST1H1D | MYC | S1PR2 | ZNF106 |
| CD79A | HIST1H1E | MYC_Promoter/5'UTR | SF3B1 | ZNF292 |
| CD79B | HIST1H2AC | MYD88 | SGK1 | ZNF296 |
| CD83 | HIST1H2AG | NAV1 | SLA | ZNF423 |
| CDC73 | HIST1H2AM | NCKIPSD | SMARCA4 |  |
| CDKN1B | HIST1H2BC | NCOR1 | SOCS1 |  |
| CDKN2A | HIST1H2BK | NFATC1 | SOX5 |  |
| CDKN2B | HIST2H2BE | NFKBIA | SPEN |  |
| CIITA | HLA-A | NFKBIE | SPIB |  |
| COL12A1 | HLA-B | NFKBIZ_3'UTR | STAT3 |  |
| COX10 | HLA-C | NLRP8 | STAT6 |  |
| CREBBP | HNRNPD | NOTCH1_last exon | SUMO2 |  |
| CTDP1 | HNRNPDL | NOTCH2_last exon | SYK |  |
